# Supplementary figures and images for: Novel cholinesterase paralogs of Schistosoma mansoni have perceived roles in cholinergic signalling and drug detoxification and are essential for parasite survival
Source: PLoS Pathog. 2019 Dec 6;15(12):e1008213. doi: 10.1371/journal.ppat.1008213 (PMC6919630; doi:10.1371/journal.ppat.1008213)

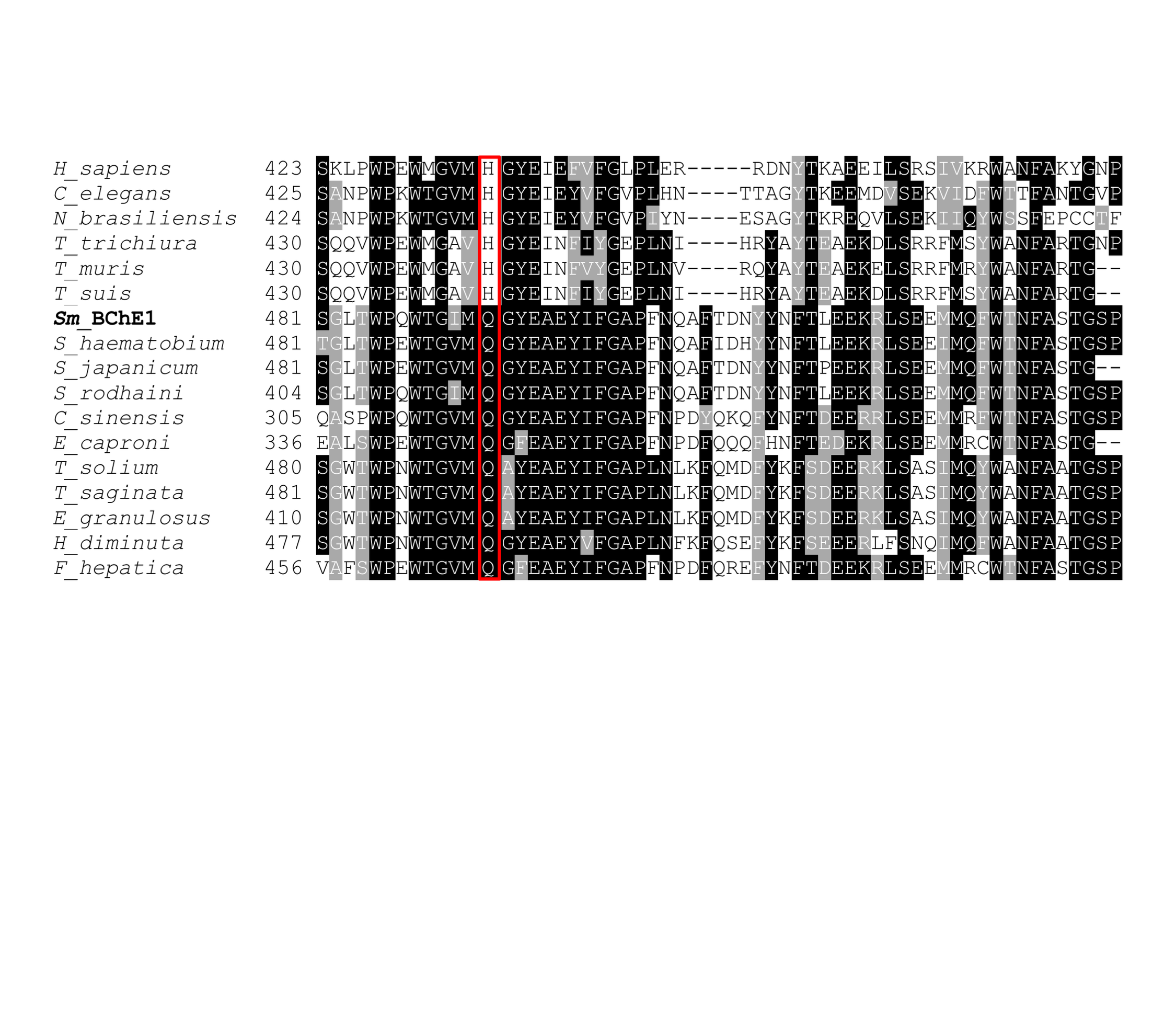

Supplement: S1 Fig — Accession numbers: Schistosoma mansoni (SmBChE1 –Smp_125350), Schistosoma rodhaini (SROB_0000329201), Schistosoma haematobium (KGB33101), Schistosoma japonicum (Sjp_0015690), Clonorchis sinensis (csin111679), Echinostoma caproni (ECPE_0000670801), Fasciola hepatica (PIS83327.1), Hymenolepis diminuta (HDID_0000005301), Echinococcus granulosus (EGR_07475.1), Taenia solium (TsM_000234300), Taenia saginata (TSAs00071g07627m00001), Trichuris muris (TMUE_3000012587), Trichuris trichiura (TTRE_0000364501), Trichuris suis (M514_03850), Nippostrongylus brasiliensis (NBR_0000102801), Caenorhabditis elegans (Y48B6A.8.1). Red box = catalytic triad residue. (TIF) [file ppat.1008213.s001.tif]

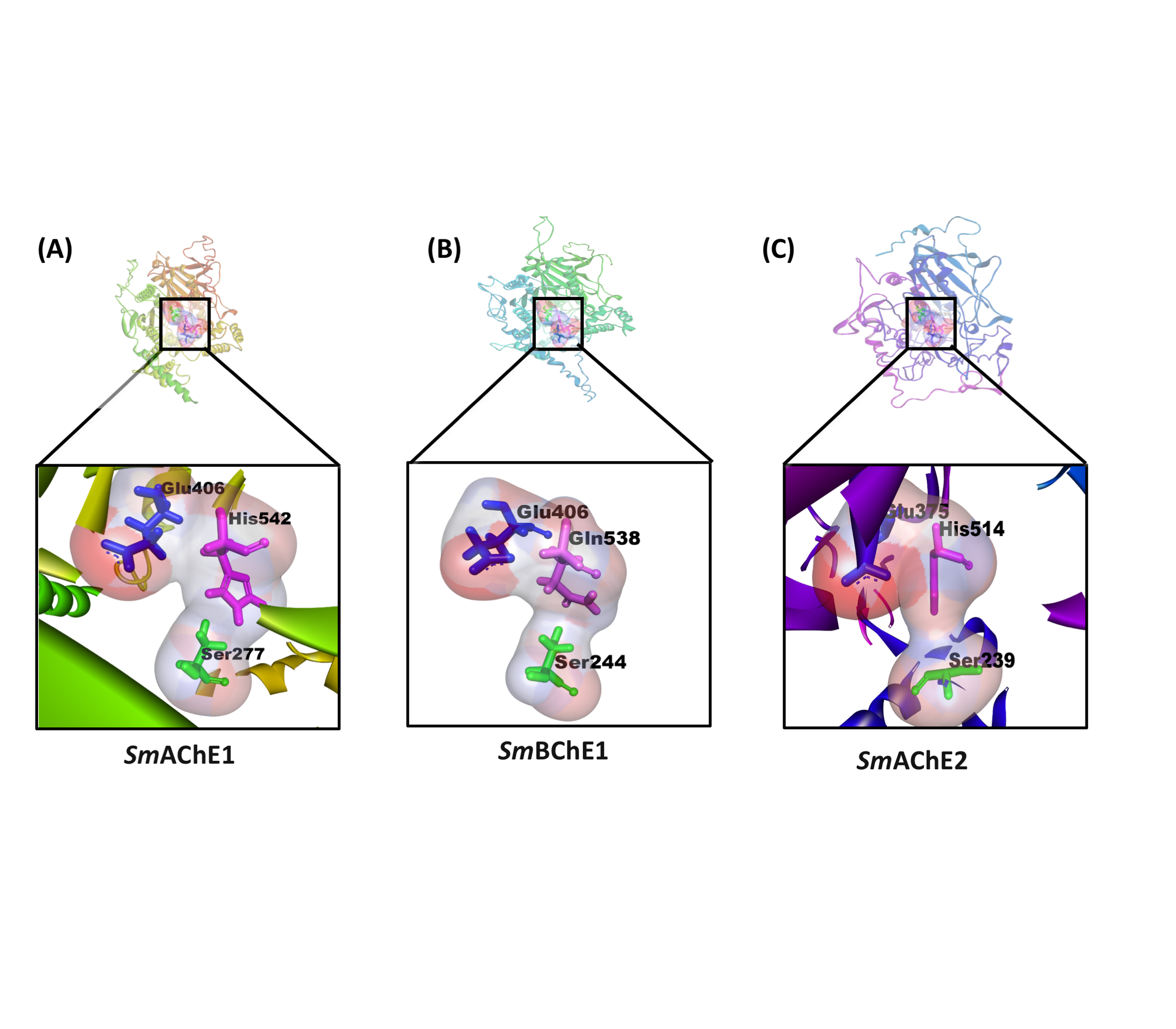

Supplement: S2 Fig — (A) SmAChE1. (B) SmBChE1. (C) SmAChE2. The amino acid residues of the catalytic triad of each paralog are magnified and their position number is given according to Torpedo AChE numbering: SmAChE1 (Ser277, His542, Glu406), SmBChE1 (Ser244, Gln538, Glu406), and SmAChE2 (Ser239, His514, Glu375). (TIF) [file ppat.1008213.s002.tif]

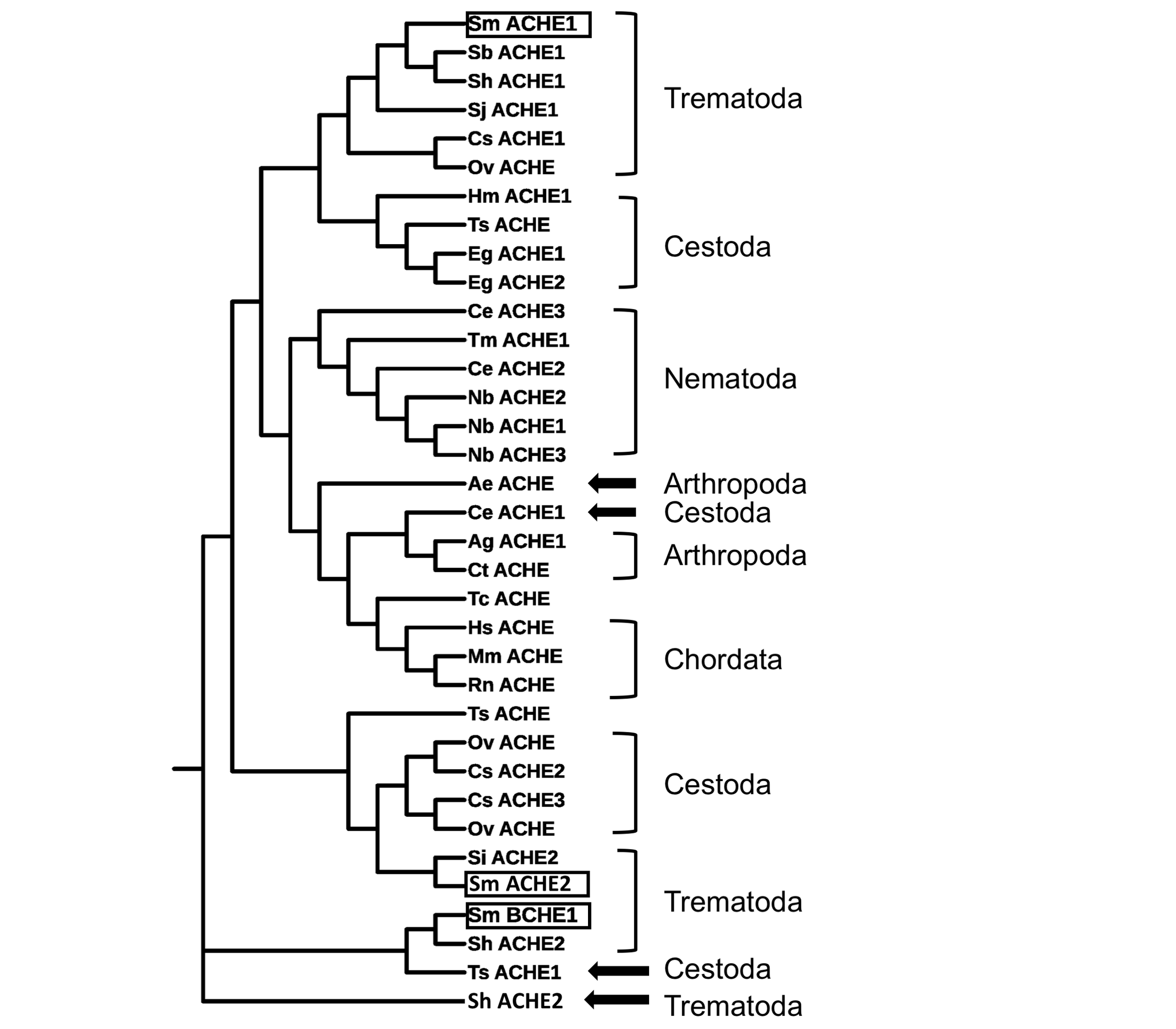

Supplement: S3 Fig — Evolutionary history was inferred using the Neighbor-Joining method and the phylogenetic tree was generated using a ClustalW alignment. The evolutionary distances were computed using the Poisson correction method and are in the units of the number of amino acid substitutions per site. All positions containing gaps and missing data were eliminated, making for a total of 236 positions in the final dataset. The three SmChEs are indicated by bold font inside a black box. Accession numbers: Schistosoma mansoni (Sm_AChE1—Smp_154600, Sm_BChE1—Smp_125350, Sm_AChE2—Smp_136690); Schistosoma bovis (Sb_AChE1—AAQ14323); Schistosoma haematobium (Sh_AChE1—AAQ14322, Sh_AChE2—KGB33101, Sh_AChE3—KGB33661); Schistosoma japonicum (Sj_AChE1—ANH56887, Sj_AChE2—Sjp0045440.1); Clonorchis sinensis (Cs_AChE1—GAA52478, Cs_AChE2—GAA53463, Cs_AChE3—GAA27255); Opisthorchis viverrini (Ov_AChE—XP009170845, Ov_AChE—XP009168237, Ov_AChE—XP009170760); Echinococcus granulosus (Eg_AChE1—JN662938, Eg_AChE2—EgG000732400); Hymenolepis microstoma (Hm_AChE1—LK053025); Taenia solium (Ts_AChE1—TsM000234300, Ts_AChE—TsM001220100, Ts_AChE—TsM000001700); Anopheles gambiae (Ag_AChE1—AGM16375); Aedes aegypti (Ae_AChE—AAB35001); Culex tritaeniorhynchus (Ct_AChE—BAD06210); Caenorhabditis elegans (Ce_AChE1—NP510660, Ce_AChE2—NP491141, Ce_AChE3—NP496963); Trichuris muris (Tm_AChE1—TMUEs0033000600); Nippostrongylus brasiliensis (Nb_AChE1—AAK44221, Nb_AChE2—AAC05785, Nb_AChE3—AAK44221); Homo sapiens (Hs_AChE—NP000656); Torpedo californica (Tc_AChE—CAA27169); Danio rerio (Dr_AChE—NP571921); Mus musculus (Mm_AChE—CAA39867); Rattus norvegicus (Rn_AChE—NP742006). (TIF) [file ppat.1008213.s003.tif]

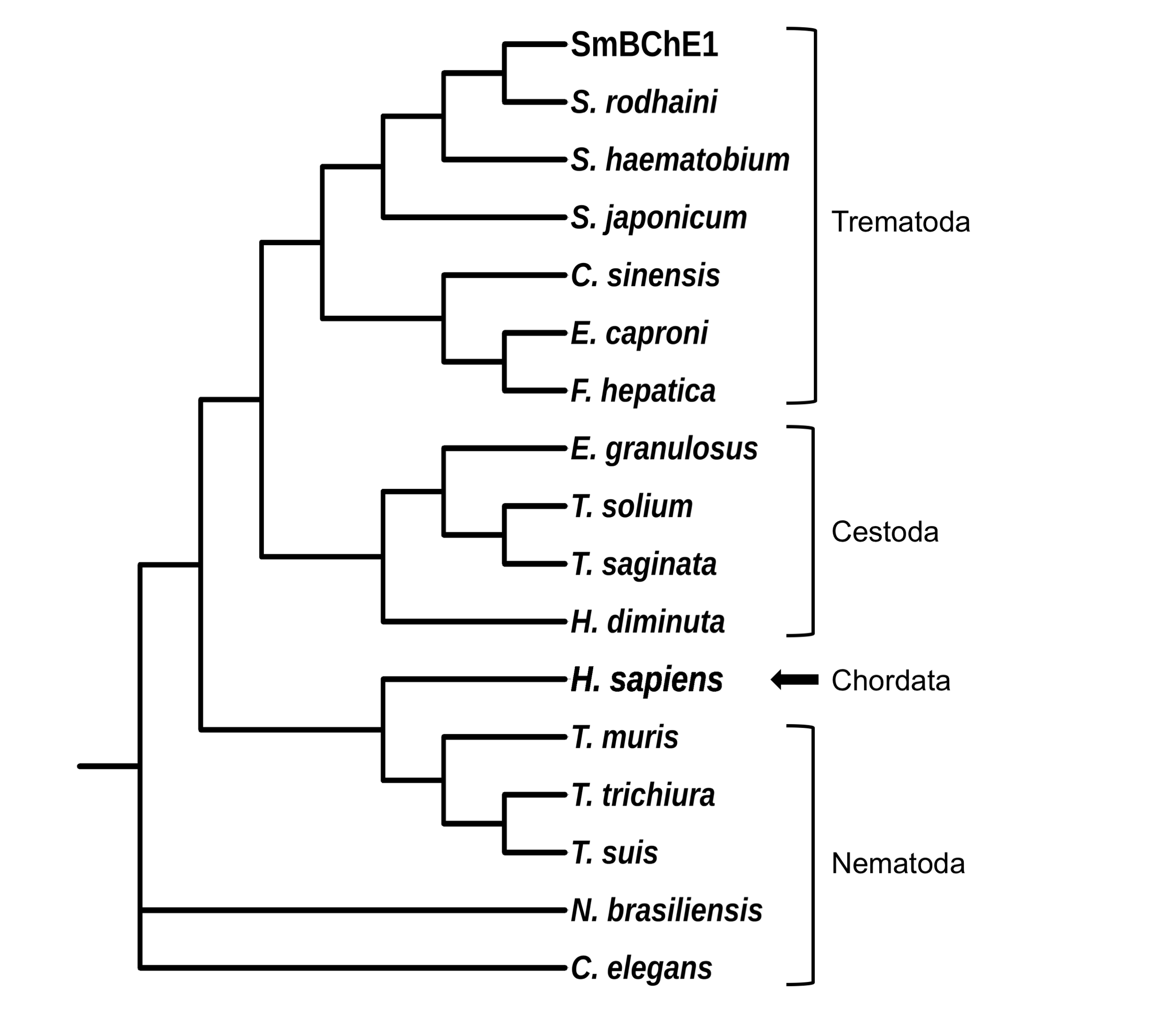

Supplement: S4 Fig — The phylogenetic tree was built using the maximum likelihood method with SmBChE1 and the top 16 helminth ChE homologs identified from the BLASTp search, as well as human BChE. Accession numbers: Schistosoma mansoni (SmBChE1 –Smp_125350), Schistosoma rodhaini (SROB_0000329201), Schistosoma haematobium (KGB33101), Schistosoma japonicum (Sjp_0015690), Clonorchis sinensis (csin111679), Echinostoma caproni (ECPE_0000670801), Fasciola hepatica (PIS83327.1), Hymenolepis diminuta (HDID_0000005301), Echinococcus granulosus (EGR_07475.1), Taenia solium (TsM_000234300), Taenia saginata (TSAs00071g07627m00001), Trichuris muris (TMUE_3000012587), Trichuris trichiura (TTRE_0000364501), Trichuris suis (M514_03850), Nippostrongylus brasiliensis (NBR_0000102801), Caenorhabditis elegans (Y48B6A.8.1). (TIF) [file ppat.1008213.s004.tif]

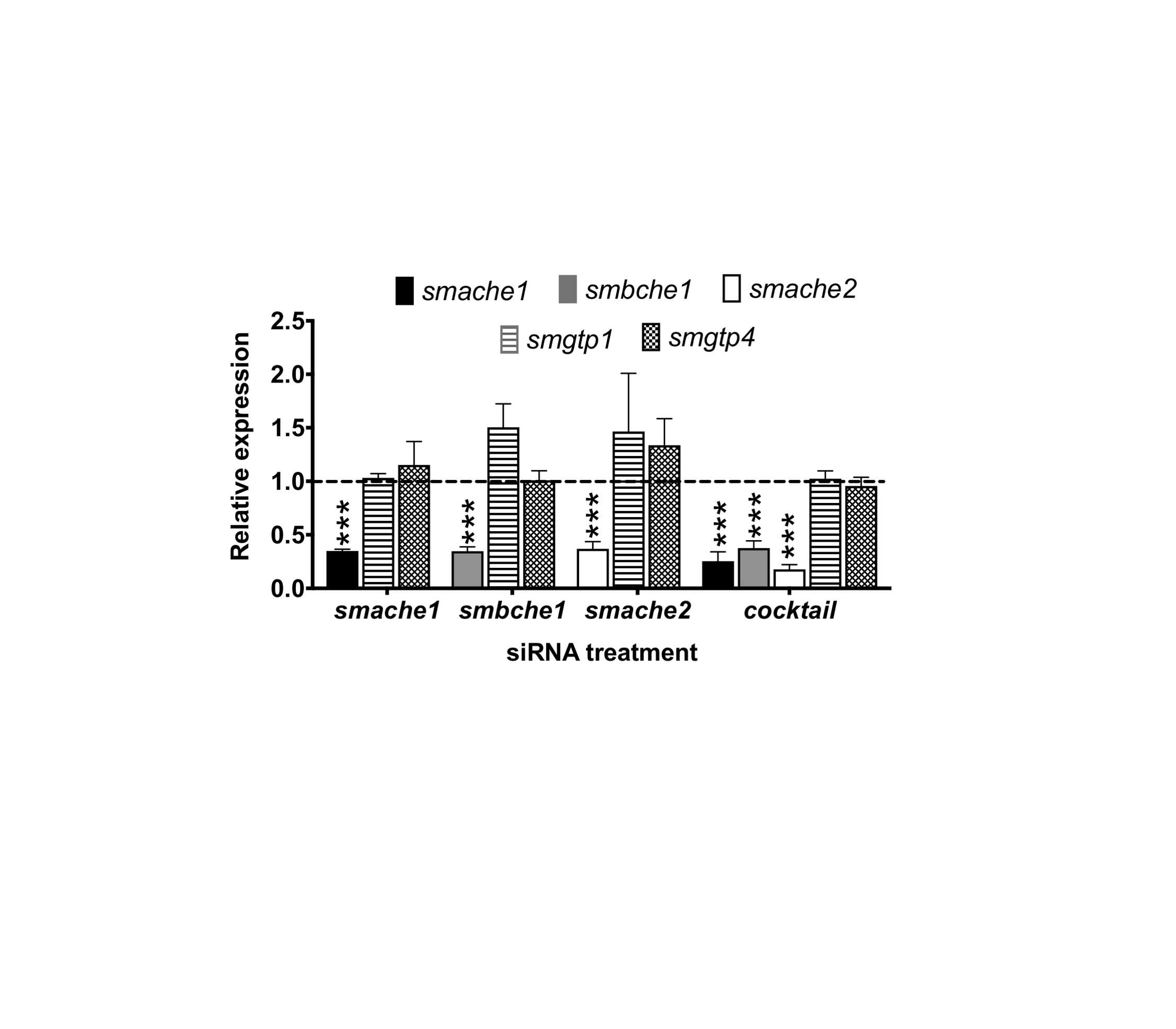

Supplement: S5 Fig — Transcript levels of each smche and sgtp in parasites treated with smche siRNAs were determined 48 h after electroporation and are shown relative to smche transcript expression in schistosomula treated with the luc control siRNA (dashed line) and represent the mean ± SEM of triplicate qPCR assays from 2 biological replicates of each treatment). Transcript expression in all parasites was normalized with the housekeeping gene, smcox1. Differences in transcript levels (relative to the luc control) were measured by the student’s t test. *P ≤ 0.05, **P ≤ 0.01, ***P ≤ 0.001. (TIF) [file ppat.1008213.s005.tif]

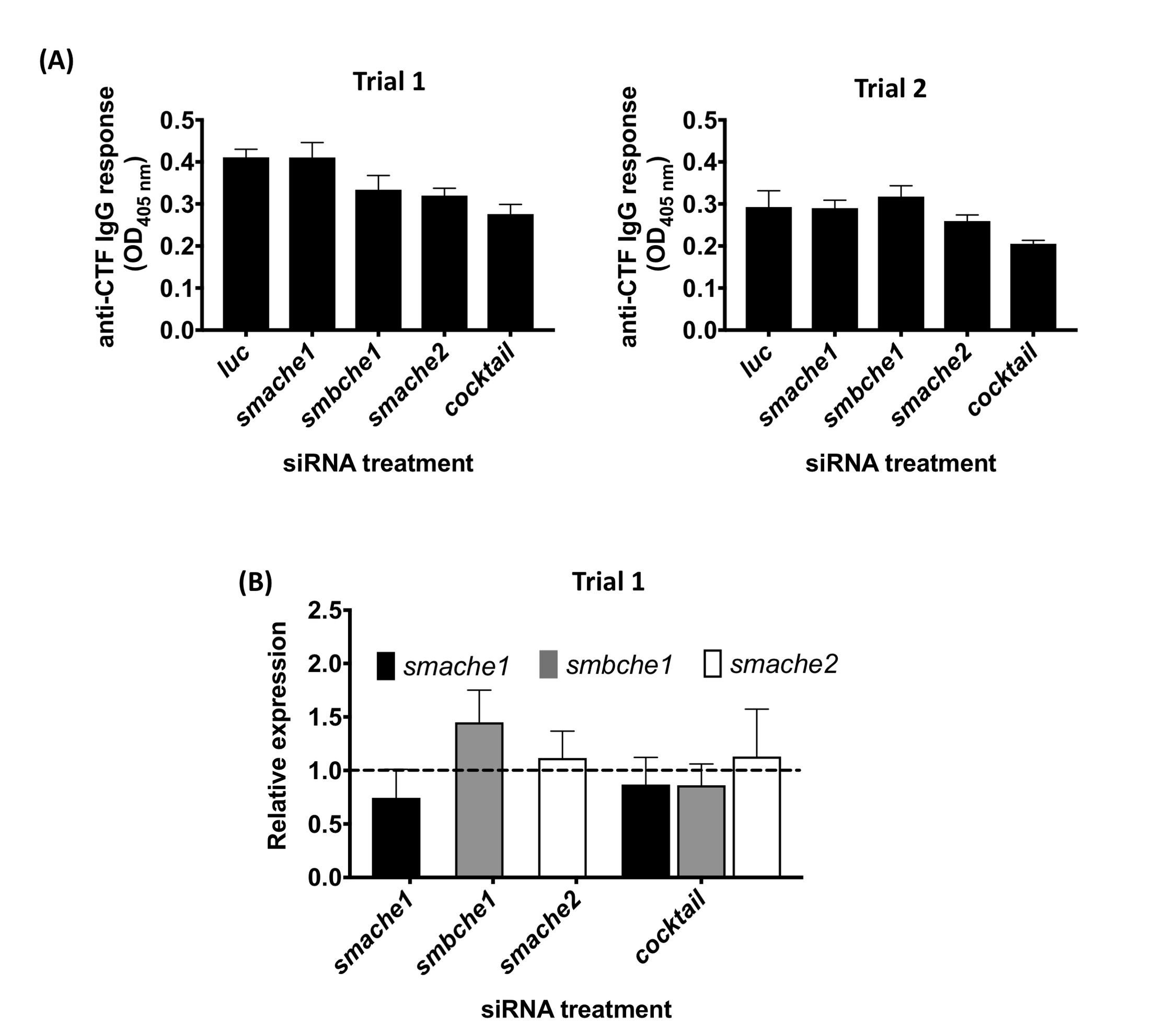

Supplement: S6 Fig — (A) For both trials, levels of serum IgG antibodies to cercarial transformation fluid (CTF) were assessed in triplicate by ELISA. Responses are shown relative to anti-CTF IgG responses of naïve mouse serum. (B) For trial 1, transcript levels of each smche in parasites recovered from necropsied mice are shown relative to smche transcript expression in schistosomula treated with the luc control siRNA (dashed line) and represent the mean ± SEM of triplicate qPCR assays. Transcript expression in all parasites was normalized with the housekeeping gene, smcox1. (TIF) [file ppat.1008213.s006.tif]

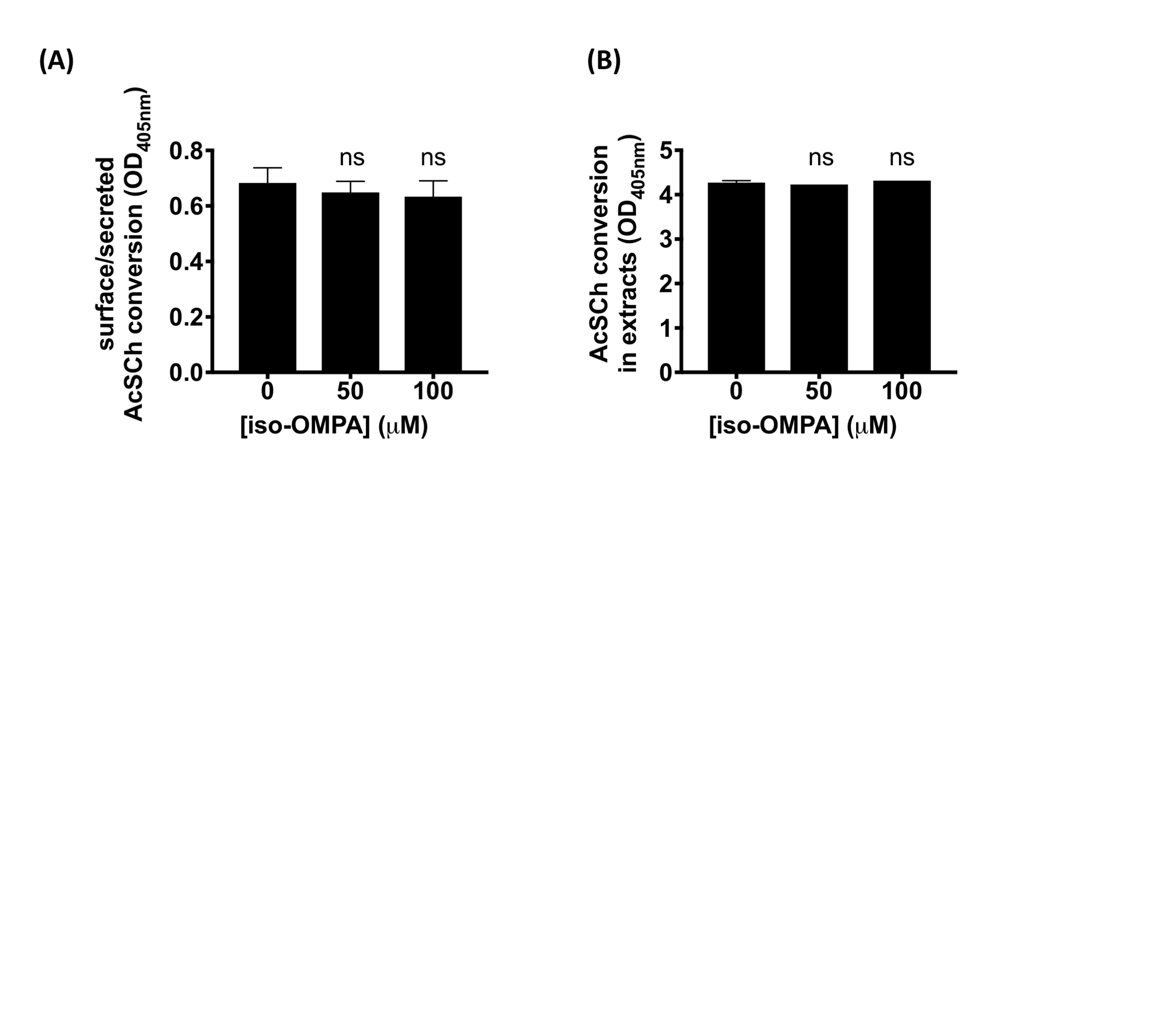

Supplement: S7 Fig — (A) Schistosomula (1000/treatment) were treated with iso-OMPA (50 and 100 μM) for 5 h and then assayed for AcSCh conversion activity. Similarly cultured, untreated parasites were used as a control. (B) Extracts were made from schistosomula from (A) and 20 μg each extract assayed for AcSCh conversion activity. Data are the average ± SEM of duplicate biological and triplicate technical experiments. Differences were measured by the student’s t test. (TIF) [file ppat.1008213.s007.tif]

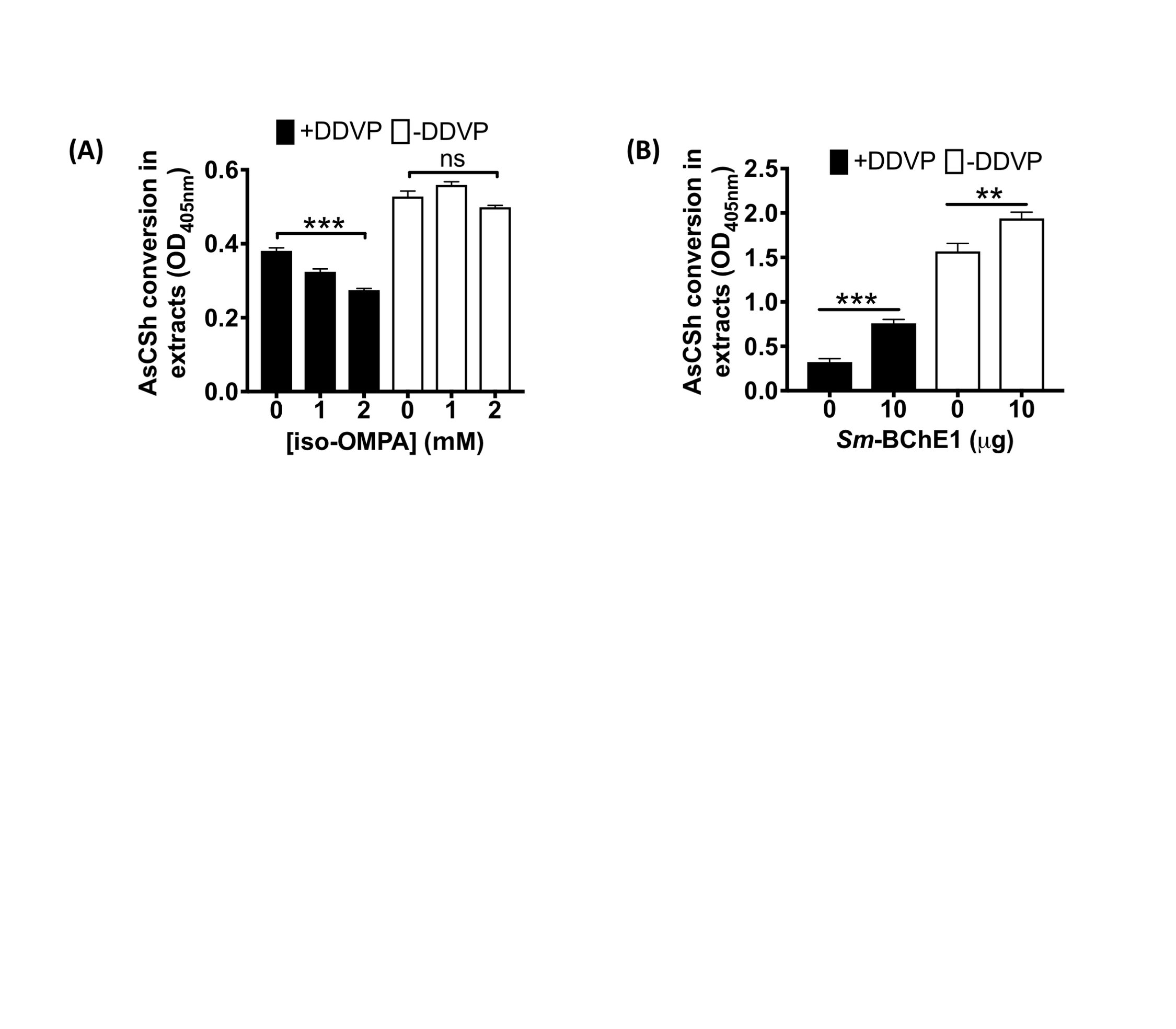

Supplement: S8 Fig — (A) Schistosomula extracts were treated with DDVP (1 μM), pretreated with iso-OMPA (1 and 2 mM) and then DDVP, before assaying AsCSh conversion activity. Identical experiments were performed in the absence of DDVP to control for AsCSh conversion activity by BChE. (B) Schistosomula extracts were pre-incubated with fSmBChE1 (10 μg) then treated with DDVP (1 μM), or treated with DDVP alone, before assaying AcSCh conversion activity. Identical experiments were performed in the absence of DDVP to control for AsCSh conversion activity by BChE. For all assays, data are the average of triplicate biological and technical experiments ± SEM and differences were measured by the student’s t test. **P ≤ 0.01, ***P ≤ 0.001. (TIF) [file ppat.1008213.s008.tif]
