# Supplementary material for: Novel cholinesterase paralogs of Schistosoma mansoni have perceived roles in cholinergic signalling and drug detoxification and are essential for parasite survival
Source: PLoS Pathog. 2019 Dec 6;15(12):e1008213. doi: 10.1371/journal.ppat.1008213 (PMC6919630; doi:10.1371/journal.ppat.1008213)
Supplement: S1 Table — (DOCX) [file ppat.1008213.s009.docx]

| **Gene** | **Direction** | **Primer Sequence** |
| --- | --- | --- |
| ***Full-length ORF expression in P. pastoris*** | | |
| ***smache1*** | Fwd | GAATTCGCGGCCGCGAATTC |
|  | Rev | TCTAGAGGTCTAGAGCTCGAG |
| ***smbche1*** | Fwd | GAATTCGCGGCCGCGAATTC |
|  | Rev | TCTAGAGGTCTAGAGCTCGAG |
| ***smache2*** | Fwd | GAATTCGCGGCCGCGAATTC |
|  | Rev | TCTAGAGGTCTAGAGCTCGAG |
| ***Partial ORF expression in E. coli*** | | |
| ***smache1*** | Fwd | GACAGAAACCACATGATGTTGGAA |
|  | Rev | TTCCAACATCATGTGGTTTCTGTC |
| ***smbche1*** | Fwd | TCCAGGAAGCACATGGTCTTCACT |
|  | Rev | AGTGAAGACCATGTGCTTCCTGGA |
| ***smache2*** | Fwd | CGCCATATGCTCTCCAAAGCGTGGTTACT |
|  | Rev | CGCCTCGAGCGGATCCCAACTTAGTCTCATC |
| ***qPCR*** | | |
| ***smache1*** | Fwd | ATGGATATGAGATTGAGTATG |
|  | Rev | CTGGAAGGATGTTAGGAT |
| ***smbche1*** | Fwd | CTACTCGTAATGATGACT |
|  | Rev | GGCTGAATTATACAAGATT |
| ***smache2*** | Fwd | ATGCGACCACACTATCACCA |
|  | Rev | CCTGATGTAAATCCACCACCA |
| ***sgtp1*** | Fwd | CTGCAGCTTATTCACTGAGTCAATC |
|  | Rev | CCACCGATGTTTTTCTGTATAACAGGAT |
| ***sgtp4*** | Fwd | AGCCAAGGAGTTAACTTATTATGCAATTTATTG |
|  | Rev | TCCAACAGATAATAACGATAACTAAAAATGGTAAGAA |
| ***smcox1*** | Fwd | TAGGGTTGGTGGTGTCACAG |
|  | Rev | ACGGCCATCACCATACTAGC |
