# Supplementary material for: Novel cholinesterase paralogs of Schistosoma mansoni have perceived roles in cholinergic signalling and drug detoxification and are essential for parasite survival
Source: PLoS Pathog. 2019 Dec 6;15(12):e1008213. doi: 10.1371/journal.ppat.1008213 (PMC6919630; doi:10.1371/journal.ppat.1008213)
Supplement: S2 Table — (DOCX) [file ppat.1008213.s010.docx]

| **Gene** | **Target sequence for siRNA duplex** |
| --- | --- |
| *smache1* | CAGGAGCTTTAATGTTTGGCA |
| *smbche1* | GTATCATCTTGTACAAAGTTTAAGA |
| *smache2* | CATCAAAACCAATTGGTAAATTACGT |
| *luc* | ACTGAGACTACATCAGCTATTCTGAT |
