# Supplementary material for: Novel cholinesterase paralogs of Schistosoma mansoni have perceived roles in cholinergic signalling and drug detoxification and are essential for parasite survival
Source: PLoS Pathog. 2019 Dec 6;15(12):e1008213. doi: 10.1371/journal.ppat.1008213 (PMC6919630; doi:10.1371/journal.ppat.1008213)
Supplement: S3 Table — (DOCX) [file ppat.1008213.s011.docx]

| **Accession number^a^** | **Score** | **Seq(Sig)^b^** | **emPAI^c^** | **Protein**  **sequences** |
| --- | --- | --- | --- | --- |
| Smp_154600.1 (*Sm*AChE1) | 15457 | 19 | 4.64 | SFKCPTINMATAVTNDYR  CPTINMATAVTNDYR  RAHTLPVYFYEFQHR  AHTLPVYFYEFQHR  TVSLPMPK  QLSDIMMTYWANFAR  TGDPNILPDGR  HVTDNLNPDDPDEITEDQLK  NPFIGWPEFR  NPFIGWPEFRNSTK  SAPANLLVSTRPR  RWYPALLQQVER |
| Smp_125350.1  (*Sm*BChE1) | 89 | 2 | 0.18 | ALGTGSWTSLEVVK  YETYSPHSVATR |

^a^ identified from Uniprot database.

^b^ number of significant, distinct sequences

^c^ exponentially modified protein abundance index
